# Heat shock protein A4 ablation leads to skeletal muscle myopathy associated with dysregulated autophagy and induced apoptosis

**DOI:** 10.1186/s12967-022-03418-3

**Published:** 2022-05-14

**Authors:** Manar Elkenani, Amal Z. Barakat, Torsten Held, Daniel Marques Rodrigues, Sherok Mobarak, Surabhi Swarnka, Ibrahim M. Adham, Belal A. Mohamed

**Affiliations:** 1 Department of Cardiology and Pneumology, Heart Center, University Medical Center Göttingen, Göttingen, Germany; 2 Institute of Human Genetics, University Medical Center Göttingen, Göttingen, Germany; 3 Biotechnology Research Institute, National Research Centre, Giza, Egypt; 4 DZHK (German Center for Cardiovascular Research), Partner Site Göttingen, Göttingen, Germany

**Keywords:** HSPs, Myopathy, Autophagy

## Abstract

**Background:**

Molecular chaperones assist protein folding, facilitate degradation of misfolded polypeptides, and thereby maintain protein homeostasis. Impaired chaperone activity leads to defective protein quality control that is implicated in multiple skeletal muscle diseases. The heat shock protein A4 (HSPA4) acts as a co-chaperone for HSP70. Previously, we showed that *Hspa4* deletion causes impaired protein homeostasis in the heart. However, its functional role in skeletal muscle has not been explored.

**Methods:**

We performed a comparative phenotypic and biochemical analyses of *Hspa4* knockout (KO) mice with wild-type (WT) littermates.

**Results:**

HSPA4 is markedly upregulated in regenerating WT muscle in vivo, and in differentiated myoblasts in vitro. *Hspa4*-KO mice are marked by growth retardation and increased variability in body weight, accompanied by 35% mortality rates during the peri-weaning period. The surviving *Hspa4*-KO mice experienced progressive skeletal muscle myopathy, characterized by increased number of muscle fibers with centralized nuclei, heterogeneous myofiber size distribution, inflammatory cell infiltrates and upregulation of embryonic and perinatal myosin heavy chain transcripts. *Hspa4*-KO muscles demonstrated an accumulation of autophagosome-associated proteins including microtubule associated protein1 light chain 3-II (LC3-II) and p62/**s**equestosome accompanied by increased number of TUNEL-positive nuclei.

**Conclusions:**

Our findings underscore the indispensable role of HSPA4 in maintenance of muscle integrity through contribution in skeletal muscle autophagy and apoptosis, which might provide a novel therapeutic strategy for skeletal muscle morbidities.

**Supplementary Information:**

The online version contains supplementary material available at 10.1186/s12967-022-03418-3.

## Background

Protein homeostasis is maintained via efficient elimination of misfolded protein aggregates by protein quality control (PQC) that utilizes a repertoire of chaperones to recognize misfolded proteins and assist their refolding or facilitate their degradation, if refolding is not possible, through either the ubiquitin–proteasome system (UPS) or the autophagy-lysosome system [1].

Compared with other cell types, PQC in muscle cell is particularly challenging because muscle proteins are in a dynamic state of synthesis and degradation in response to mechanical stress. Making it worse, muscles are post-mitotic, and therefore not able to dilute toxic effect of the protein aggregates by division and, thus, are highly susceptible to misfolded proteins. Maintained PQC is critical for proper skeletal muscle homeostasis, and inefficient PQC leads to accumulation of protein aggregates and eventually to muscular disorders [2].

Autophagy is an evolutionarily conserved and a tightly regulated intracellular process that targets the misfolded proteins and damaged organelles for lysosomal degradation. Basal constitutive autophagy is required for maintaining muscle function [3]. Excess attenuation or augmentation of the autophagy result in muscle morbidities [4–7].

Heat shock proteins (HSPs) function as molecular chaperones to maintain cellular PQC through mediating efficient protein folding and targeting misfolded protein aggregates for degradation, and therefore have an indispensable role for proper myogenesis [8–12]. Mutations in human HSPs have been identified in patients with muscle myopathy [13–15]. HSPA4 belongs to HSP110 family that functions as a co-chaperone for HSP70 [16]. HSPA4 is ubiquitously expressed [17], and has been shown to avert inflammation and apoptosis, protect from oxidative stress and improve survival [18–20]. A role of HSPA4 in the cross talk between UPS and autophagy has been proposed, but there was no proof for this hypothesis [21]. *Hspa4*-knockout (KO) mice showed impaired PQC in the heart, characterized by accumulation of misfolded protein aggregates, and resulting in pathological myocardial remodeling and fibrosis [22]. Given the fundamental importance of PQC in skeletal muscle, we hypothesized that HSPA4 would be a novel regulator in skeletal muscle homeostasis.

Here, we observed that *Hspa4*-KO mice exhibit decreased survival rates, growth retardation and increased variability in body weight. The aged *Hspa4*-KO mice develop spinal deformities and kyphosis. We therefore characterized the skeletal muscles in *Hspa4*-KO mice and showed that HSPA4 deficiency causes skeletal muscle myopathy associated with dysregulated autophagy and enhanced apoptosis.

## Methods

### Animals

Male and female *Hspa4*-KO mice were generated on 129/Sv genetic background as described previously [17].

### Western blot analysis

Protein lysates were extracted from frozen tibialis anterior (TA) muscles using RIPA lysis buffer (Millipore) containing protease and phosphatase inhibitor cocktail (Roche Diagnostics). Aliquots of 20 μg lysates were resolved on a NuPage 4–12% SDS-PAGE. Western blotting was carried out using the following primary antibodies: rabbit anti-LC3, anti-p62 (Cell signaling technology), anti-BCL-2 (Abcam), anti-HSPH1 (Sigma Aldrich), anti-HSPA4L, anti-HSPA4, mouse anti-BAX and anti-GAPDH (Santa Cruz Biotechnology). For quantification, an enhanced chemiluminescence detection system (Amersham Bioscience) and Image Lab software (Bio-Rad) were used according to the manufacturer’s instructions.

### Histological analyses

Muscles were collected and either paraffin-embedded, or immediately frozen in isopentane. Sections (6 µm) were stained with hematoxylin and eosin (H&E), and the number of centrally nucleated fibers was counted across 5 separate fields of view from at least three sections of each mouse. TUNEL assay was performed in paraffin-embedded sections using In Situ Cell Death Detection Kit (Roche Diagnostics). After fixation in ethanol–acetic acid, TA sections were treated with proteinase K and permeabilized with 0.5% Triton X-100. The sections were then incubated in the TUNEL reaction mixture containing terminal deoxynucleotidyl transferase and nucleotide mixture for 60 min at 37 °C in a dark humid chamber. TUNEL-positive cells were counted in 5–8 random fields/ muscle. For immunofluorescence, frozen sections were permeabilized using 0.2% Triton X-100 in phosphate-buffered saline (PBS), blocked with 5% bovine serum albumin in PBS and incubated with rabbit anti-LC3 (Cell signaling technology). Photomicrographs were captured using a microscope Olympus BX60 fluorescence microscope.

### Quantitative real-time polymerase chain reaction (qRT-PCR) and Northern blotting

For real time PCR, cDNA synthesis was carried out with iScript cDNA synthesis kit (Bio-Rad). QRT-PCR was performed on a Biorad iQ-Cycler using SYBR Green Supermix (Bio-Rad). For Northern blot analysis, 20 μg of total RNA samples was size fractionated by electrophoresis, transferred onto nylon membrane (Amersham Bioscience) and hybridized with a 32P-labeled fragments. All the primers used are listed in the Additional file 1: Table S1.

### Cell culture

Mouse C2C12 myoblasts [American Type Culture Collection (ATCC)] were cultured in growth media (GM) containing Dulbecco’s modified Eagle’s medium (DMEM) (Invitrogen), 10% fetal bovine serum and 1% penicillin–streptomycin (Sigma-Aldrich). Differentiation in C2C12 cultures was induced by replacing the growth with differentiation medium (2% horse serum in DMEM and 1% antibiotic mixture).

### Determination of 20S proteasome activity

Using 20S Proteasome Assay Kit (10,008,041; Biomol), the 20S proteasome assay was carried out in a total volume of 100 μl in 96 well plates. Assays were initiated by addition of 100 μM of fluorescently labeled substrate, succinyl-Leu-Leu-Val-Tyr-7-amido-4-methylcoumarin (Suc-LLVY-AMC), to the protein lysates (50 μg) and incubation at 37 °C. These substrates are cleaved by the proteasome, releasing free AMC which was then measured spectrofluorometrically after one hour at an excitation wavelength of 360 nm and an emission wavelength of 480 nm. Each assay was conducted in duplicates and in the absence and presence of the specific proteasomal inhibitor, lactacystin (20 μM).

### Cardiotoxin (CTX) injection

Adult mice were anesthetized with isoflurane and 50 μL of 10 μM cardiotoxin was injected into the left TA muscle. As a control 0.9% saline (vehicle) was injected in the contralateral side. Carprofen was used for post-treatment analgesia. Mice were sacrificed and TA muscles were dissected at various time points after injection.

### Statistical analysis

Statistical analysis was carried out using GraphPad Prism 7.0 (GraphPad Software, Inc, California, USA) with two-tailed unpaired Student’s t-test or one-way ANOVA with Bonferroni post-test correction where appropriate. Kaplan–Meier survival analysis was performed, and a Log-rank test was used to determine significance.

## Results

### Upregulation of HSPA4 during skeletal muscle regeneration and myoblast differentiation

**Fig. 1:**
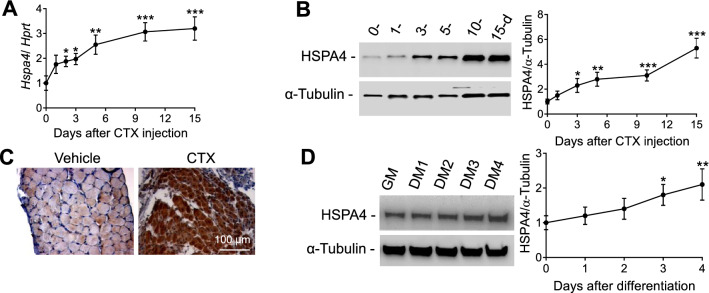
Up-regulation of HSPA4 in regenerating myocyte and differentiating myoblast. **A** mRNA expression of *Hspa4*, evaluated by real time PCR, in tibialis anterior after cardiotoxin injection (*n* = 3/time point). **B** Western blotting of HSPA4 protein after cardiotoxin injection (left panels) and densitometry measurement (right panel) (*n* = 3/time point). **C** Representative immunostaining of WT tibialis anterior sections for HSPA4 protein at 5 days after vehicle- and cardiotoxin-treatment. **D** Immunoblots (left panels) and densitometry analysis (right panel) of HSPA4 levels in C2C12 during proliferation in growing medium (GM) and differentiation for 1–4 days in differentiation medium (DM1-DM4) (*n* = 3/ time point). Data are expressed as mean ± SEM. *p < 0.05, **p < 0.01, ***p < 0.001 vs. corresponding controls, two-tailed unpaired Student's t-test

Western blot analysis revealed that HSPA4 is ubiquitously expressed in different skeletal muscles Additional file 1: Fig. S1). Markedly increased *Hspa4* mRNA and protein levels were detected in regenerating TA muscle after CTX injection, which induces muscle degeneration followed by regeneration (Fig. 1A–C). HSPA4 protein expression was also markedly upregulated in immortalized C2C12 myoblasts after induction of differentiation (Fig. 1D). Taken together, our data suggest a relevant role of HSPA4 in myogenesis, which prompted us to characterize skeletal muscles in *Hspa4*-KO mice.

### Delayed growth and early mortality in Hspa4-KO mice

**Fig. 2:**
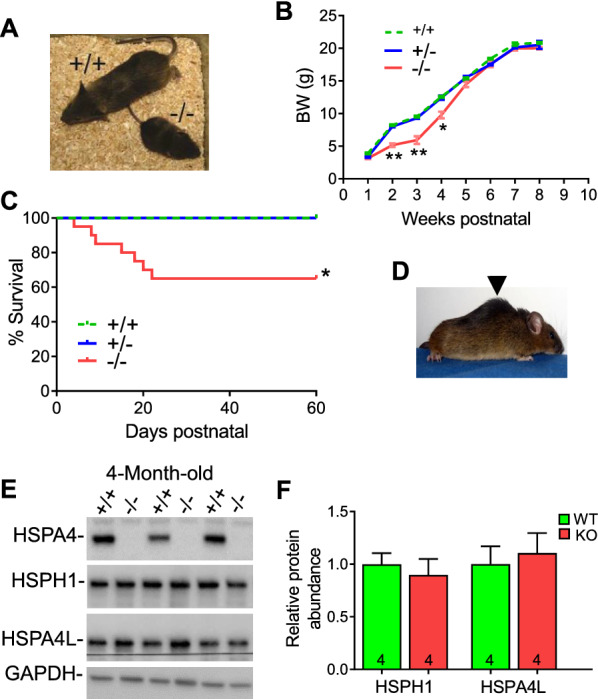
Stunted growth and early peri-weaning mortality in *Hspa4*-KO mice. **A** Gross view of 2-week-old *Hspa4*-KO and sex-matched WT littermate. **B** Growth curve of WT and *Hspa4*-KO mice (*n* = 5/genotype/age). **C** Survival probability of WT and *Hspa4*-KO mice, evaluated by Kaplan–Meier curves. *p < 0.05 vs. WT, log-rank test. **D** Representative photograph of 12-month-old *Hspa4*-KO mice (Arrowhead indicates kyphosis). **E** and **F**, Immunoblots **E** and densitometry analysis **F** of HSPs levels in tibialis anterior from 4-month-old mice. Data are expressed as mean ± SEM. *p < 0.05, **p < 0.01 vs. WT controls, two-tailed unpaired Student's t-test

*Hspa4*-KO pups were born from heterozygous breeding pairs at the expected Mendelian ratios (Table 1). In the first week postnatal, the *Hspa4*-KO pups were indistinguishable from their *Hspa4*^+/+^ and *Hspa4*^±^ littermates. However, starting at postnatal day 8 (P8), *Hspa4*-KO pups gained less weight and showed clear growth retardation between P14 and P28 (Fig. 2A, B), likely the result of decreased milk intake caused by muscle weakness. Serum glucose levels and expression of the key gluconeogenic enzyme, lipid transport related-genes and growth hormone-responsive gene did not show any significant difference between *Hspa4*-KO and control animals (Additional file 1: Fig. S2). In accordance with stunted growth, *Hspa4*-KO mice also exhibited generalized dwarfism affecting all organs tested. However, the decrease in muscle mass was much more severe than other organs (Table 2). About 35% of the *Hspa4*-KO mice died during the peri-weaning period (Fig. 2C). After weaning, surviving *Hspa4*-KO mice normalized their body weight and were generally similar to wild-type (WT) littermates by two months of age (Fig. 2B). By the age of 12 months, *Hspa4*-KO mice developed spinal deformity in the form of kyphosis (Fig. 2D), indicative of paraspinal muscles weakness [23]. The protein level of HSPA4L and HSPH1, other members of HSP110 family, was not different between WT and *Hspa4*-KO muscles ruling out any compensatory upregulation of the studied proteins in the *Hspa4*-KO muscles (Fig. 2E, F).

**Table 1:** *Hspa4*-KO mice were born at expected Mendelian ratios

*Hspa4* genotype (112 mice)	Predicted	Observed
*Hspa4*^+/+^	28 (25%)	29 (≈26%)
*Hspa4*^±^	56 (50%)	59 (≈53%)
*Hspa4*^−/−^	28 (25%)	24 (≈21%)

**Table 2:** Growth retardation associated with decreased muscle mass in 2-week-old *Hspa4*-KO mice

	WT (*n* = 6)	*Hspa4*-KO (*n* = 6)	% Control
BW (g)	9.58 ± 0.40	5.37 ± 0.59^**^	56
Tibia length (mm)	10.67 ± 0.33	8.5 ± 0.29^**^	80
TA (mg)	19.33 ± 1.76	8.5 ± 1.5^**^	44
Quadriceps (mg)	31 ± 0.58	16.25 ± 3.07^*^	52
Gastrocnemius (mg)	25 ± 0.58	11.75 ± 2.21^**^	47
Heart (mg)	50.33 ± 2.03	37.00 ± 5.74	74
Kidney (mg)	50.33 ± 2.65	39.25 ± 2.56^*^	79

Data are presented as mean ± SEM. *p < 0.05, **p < 0.01 vs. WT, two-tailed unpaired Student's t-test

### Skeletal muscle myopathy in Hspa4-KO mice

**Fig. 3:**
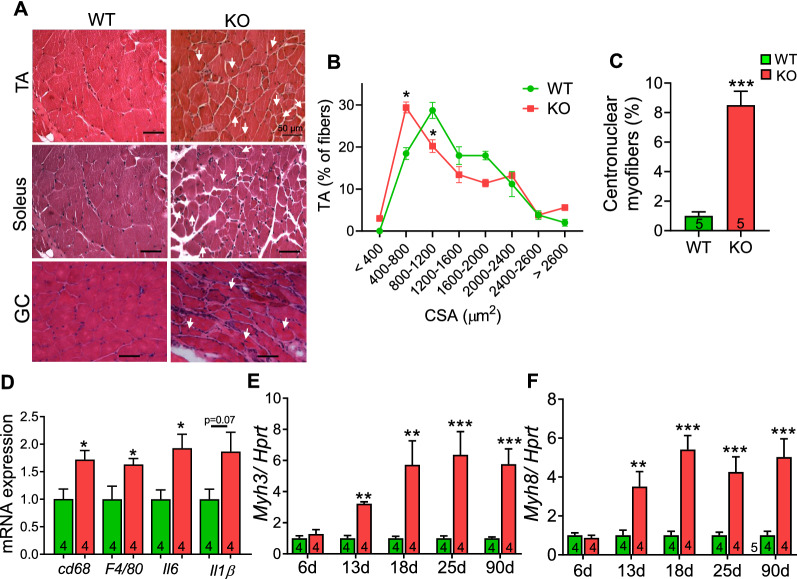
Skeletal muscle myopathy in 4-month-old *Hspa4*-KO mice. **A** Representative sections from tibialis anterior (TA), soleus and gastrocnemius (GC) muscles, stained with H&E showing centrally nucleated myofibers (white arrows). **B** and **C** Fiber size distribution in tibialis anterior (*n* = 4 mice/genotype; 250 fibers/ mouse) **B**, and average percentage of myofiber with central nucleus **C**. **D–F** Quantitative real time PCR analysis of inflammatory markers **D**, *Myh3* **E** and *Myh8* **F** expression. Data are expressed as mean ± SEM. *p < 0.05, **p < 0.01, ***p < 0.001 vs. WT controls, two-tailed unpaired Student's t-test. Numbers within columns indicate mice

By the age of 4 months, H&E-stained *Hspa4*-KO TA muscles exhibited myopathic changes including heterogeneous myofiber size distribution, numerous centrally nucleated fibers (CNF), and inflammatory cell infiltrates (Fig. 3A–C). The induction of the inflammatory response in *Hspa4*-KO TA muscles was confirmed at the transcript level by the detection of macrophage-specific markers, *Cd68* and *F4/80*, and interleukins, *Il6* and *Il1β* (Fig. 3D). TA muscles from *Hspa4*-KO mice experienced induction of embryonic (*Myh3)* and perinatal (*Myh8)* muscle myosin heavy chain genes, indicative of ongoing de- and regeneration (Fig. 3E, F). These data suggest that the *Hspa4* deletion impairs the integrity of the myofibers resulting in the activation of a regenerative response. Interestingly, induction of *Myh8* and *Myh3* and increased percentage of CNF were also observed in *Hspa4*-KO muscles during the peri-weaning period (Fig. 3E, F and Additional file 1: Fig. S3). Myopathy was observed in other examined muscles including soleus, gastrocnemius and paraspinal muscles (Fig. 3A and Additional file 1: Fig. S4), indicating a generalized skeletal muscle involvement in *Hspa4*-KO mice.

### Preserved skeletal muscle regeneration in Hspa4-KO mice

**Fig. 4:**
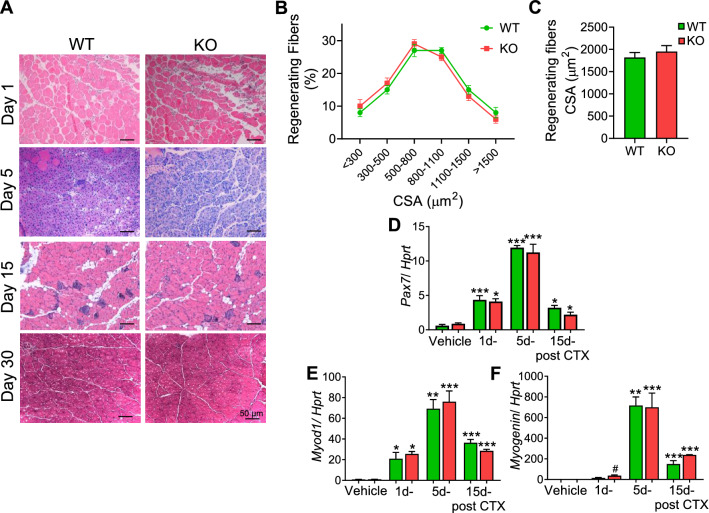
Similar regeneration efficiency in WT and *Hspa4*-KO muscles. **A** Morphological assessment of injured tibialis anterior muscles was performed via H&E staining post-injury. **B** Graph illustrating the distribution of regenerating myofiber size frequency. **C** Average CSA of regenerating fibers containing centralized nuclei. **D–F** Real time PCR analyses of the myogenic transcription factors in injured muscles. Data are expressed as mean ± SEM. *p < 0.05, **p < 0.01, ***p < 0.001 vs. corresponding vehicle, one-way ANOVA with Bonferroni post-test (*n* = 3–4mice/ time point/ genotype)

Induced HSPA4 expression in muscles following muscle injury forced us to address the requirement of HSPA4 for normal muscle regeneration and recovery following muscle injury. We monitored skeletal muscle repair in CTX-injected TA muscles of 4-month-old WT and *Hspa4*-KO mice. One day following CTX injury muscles from both genotypes showed significant fiber degeneration and necrosis, as confirmed by eosinophilic staining and marked mononuclear cell infiltration. By 5 days following injury, degenerating muscle fibers were largely cleared in WT and *Hspa4*-KO muscles, and replaced by centrally nucleated nascent myoblasts and other mononuclear cells, indicative of the early phase of muscle regeneration. At 15 days of injury, the nascent myofibers in both genotypes were similarly enlarged (Fig. 4A–C). Complete restoration of the muscle architecture was observed in both genotypes at 30 days post-injury (Fig. 4A). The relative mRNA levels of *Pax7*, Myo*d*1 and Myog*enin*, satellite cell-related markers for activation, fusion and differentiation, respectively, were not statistically different between WT and *Hspa4*-KO muscles at any time point post-injury (Fig. 4D–F). These data therefore suggest that the ability of *Hspa4-*KO mice to activate the myogenic program is not markedly altered.

Overall, our results denote that increased degeneration, rather than defective regeneration, is the underlying cause for skeletal muscle myopathy in *Hspa4*-KO mice.

### Dysregulated autophagy in Hspa4-KO muscles

**Fig. 5:**
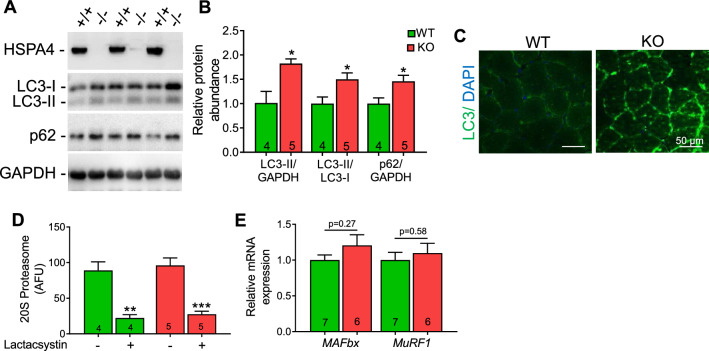
Dysregulated autophagy in *Hspa4*-KO muscles. **A** and **B** Representative Western blots **A** and densitometry analyses **B** of the expression of HSPA4, LC3-I, LC3-II and p62 proteins in 4-month-old tibialis anterior. **C** Immunofluorescent staining of frozen sections of tibialis anterior for LC3 (green) and nuclei (blue) at 4 months of age. **D** Average chymotrypsin activity in 4-month-old tibialis anterior muscles. **E** Real time PCR analyses of the atrogenes in tibialis anterior muscles from 4-month-old mice. Data are expressed as mean ± SEM. *p < 0.05, **p < 0.01, ***p < 0.001 vs. WT controls, two-tailed unpaired Student's t-test. Numbers within columns indicate mice

Autophagy is initiated with the sequestration of cytoplasmic components by isolation membrane that expands to form double-membrane vesicles, the autophagosomes, which fuse with endosome/lysosome, followed by lysosomal hydrolysis of sequestered cytoplasmic components [24]. Autophagy can be assessed by detection of the modification of microtubule associated protein1 light chain 3 (LC3) from free mature LC3-I form to membrane-bound lipidated LC3-II form and by assessment of protein level of the autophagy adaptor, p62/sequestosome [25]. *Hspa4*-KO TA muscles showed marked increased LC3-II and p62 protein levels (Fig. 5A, B). Consistently, immunofluorescence revealed an abundant LC3 puncta in *Hspa4*-KO muscles (Fig. 5C), suggesting the dysregulated autophagy as a possible cause for skeletal muscle myopathy in *Hspa4*-KO mice.

Because an impaired proteasome pathway can also lead to an accumulation of p62 protein, we assessed proteasome activity in WT and *Hspa4*-KO TA muscles. No marked changes were found in chymotrypsin enzyme activity in total homogenates from WT and *Hspa4*-KO TA muscles (Fig. 5D). Treatment with known 20S proteasome inhibitors, lactacystin, resulted in a significant, but comparable, inhibition of 20S activity in WT and *Hspa4*-KO muscles (Fig. 5D). Moreover, the expression of atrogenes, *MuRF1* and *MAFbx*, was not different between WT and *Hspa4*-KO muscles (Fig. 5E), indicating that the proteasome activity is not impaired in *Hspa4*-KO muscles.

### Induced apoptosis in Hspa4-KO muscles

**Fig. 6:**
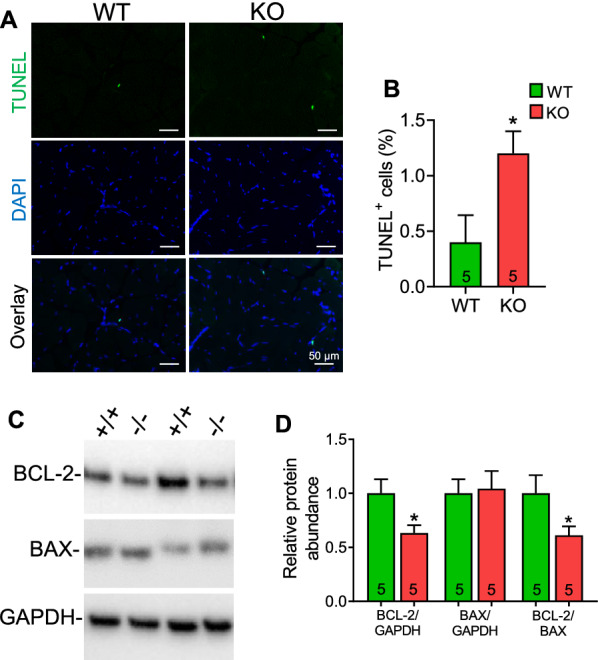
Increased apoptosis in 4-month-old *Hspa4*-KO muscles. **A** Apoptosis-positive myonuclei were detected in tibialis anterior muscles by TUNEL analysis. **B** Quantification of TUNEL-positive nuclei. **C** and **D** Western blots for the expression of BCL-2 and BAX **C** and densitometry analyses **D** Data are expressed as mean ± SEM. *p < 0.05 vs. WT, two-tailed unpaired Student's t-test. Numbers within columns indicate mice

An anti-apoptotic effect of HSPA4 was previously reported [18–20]. We examined the apoptosis in the skeletal muscle of *Hspa4*-KO mice by performing TUNEL assay using TA sections from 4-month-old WT and *Hspa4*-KO mice. The number of apoptotic nuclei was markedly increased in *Hspa4*-KO compared to WT muscles (Fig. 6A, B). Additionally, the protein level of anti-apoptotic factor BCL-2 and consequently BCL-2/ BAX ratio were markedly decreased in *Hspa4*-KO TA muscles, suggesting that apoptosis may have exacerbated skeletal muscle myopathy in *Hspa4*-KO mice.

## Discussion

Here, we identified a previously unexplored role of HSPA4 in skeletal muscle homeostasis using *Hspa4*-KO mice. Previous reports have linked HSPA4 with several morbidities and mortality [17–22]. However, its role in skeletal muscle remains unknown. Our results demonstrated that HSPA4 is ubiquitously expressed in all muscles tested including fast twitch (TA and gastrocnemius) and slow twitch (soleus) muscles. Furthermore, HSPA4 expression is induced in regenerating WT muscles upon CTX-induced muscle injury and in myoblast upon differentiation, highlighting a potential role of HSPA4 in myogenesis.

Our results showed that HSPA4 is crucial for normal survival and growth. *Hspa4*-KO mice show growth retardation associated with 35% mortality rates within the first 4 weeks of life, which is likely due to skeletal muscle affection. Although cardiac structure and function are not deteriorated at the peri-weaning stage in *Hspa4*-KO mice [22], we cannot exclude acute decompensated myocardial function, beside skeletal muscle myopathy, as a possible underlying cause of early death in *Hspa4*-KO mice. *Hspa4*-KO mice that survive the first month of life develop a progressive myopathy, characterized by centrally nucleated myofibers, heterogeneous myofiber size distribution and inflammatory cell infiltrates, associated with defective autophagy and increased apoptotic cell death.

The UPS and autophagy are the major proteolytic systems of the cell that have a crucial role in the removal of protein aggregates. As one of the post-mitotic tissues, the highly dynamic skeletal muscle is particularly vulnerable to dysfunctional organelles and aggregation-prone proteins. In this regard, it is not surprising that dysregulated activity of the autophagy and/or UPS is implicated in a variety of myofiber degeneration and muscle weakness [5, 26]. Several molecular chaperones and co-chaperones, including HSPA4, play a role in the cross-talk between UPS and autophagy to maintain cellular protein homeostasis [21, 27, 28].

Autophagy is markedly dysregulated in *Hspa4*-KO muscles as shown by accumulation of LC3-II protein. Thus, it is tempting to speculate that perturbed autophagy contribute to the muscle abnormalities in *Hspa4*-KO mice. However, increased LC3-II protein level can occur due to either induction of early or inhibition of late autophagy. We therefore examined the p62 protein level to clarify the effect of *Hspa4* deletion on autophagy. The protein p62 is a specific target of the autophagy degradation. Thus, intracellular accumulation of this protein is indicative of insufficient autophagy [29]. Indeed, an increased p62 protein level was detected in *Hspa4*-KO muscle despite the increase of LC3-II, suggesting a late block in autophagy occurring after autophagosome formation, and involves autophagsome/ lysosome fusion or lysosomal degradation. However, autophagy is a highly dynamic and complex process, and therefore accurate assessment of the autophagy flux using lysosomal inhibitors, such as bafilomycin or chloroquine, among others, is necessary to confirm our assumption. Collectively, these data suggest that HSPA4 may have a beneficial role in the muscle via maintaining proper autophagy.

P62 is an autophagy receptor of ubiquitinated proteins that interact simultaneously with LC3 and promote the degradation of ubiquitinated protein aggregates [29]. However, no significant changes in the content of ubiquitinated proteins was found between *Hspa4*-KO and WT muscles [22], suggesting that *Hspa4* deletion in skeletal muscle does not impair the degradation of ubiquitinated proteins, despite of the accumulation of p62. Consistently, *Hspa4*-KO muscles did not exhibit perturbed UPS activity, as evidenced by comparable proteasome activity and atrogenes expression to that in WT muscles.

Autophagy is an essential protective mechanism against apoptotic cell death [30]. Moreover, anti-apoptotic effect of HSPA4 has been previously reported [18–20]. Our results consistently revealed a significantly higher proportion of TUNEL-positive nuclei, downregulation of anti-apoptotic BCL-2 in the *Hspa4*-KO muscles, indicating that increased apoptosis, probably due to impaired autophagy, may be one of the reasons for the skeletal myopathy observed in *Hspa4*-KO muscles.

The transcription factor nuclear factor κB (NF-κB) is a key mediator of inflammation through induction of various pro-inflammatory cytokines, including interleukins and a large number of inflammatory genes, including macrophages-related markers [31]. Recently, it has been reported that HSPA4 inactivates NF-κB pathway and therefore inhibits inflammatory signaling [19]. Consistently, we showed here that the expressions of *Il1b* and *Il6* as well as *Cd68* and *F4/80* are increased in *Hspa4*-KO muscles, suggesting an overall inflammation, possibly due to augmented NF-κB activity. However, a comprehensive analysis of inflammation in our mice is needed to support this hypothesis.

Several genes are associated with inherited skeletal muscle myopathies, and the list is still expanding [32]. Although *Hspa4* mutations have not yet been linked to any muscle morbidities in human, the myocardium of *Hspa4*-deficient mice experiences pathological remodeling and fibrosis [22], which highlights the importance of HSPA4 for striated muscle integrity, and suggests that HSPA4 may be a promising therapeutic candidate for skeletal muscle myopathy. It remains to be addressed whether myopathy patients with genetically unknown cause carry *Hspa4* mutations*.* We therefore propose that genetic screening by *Hspa4* gene sequencing could identify novel mutations and expand the spectrum of myopathy-associated genes in patients with inherited skeletal muscle myopathies and/or pediatric heart diseases.

A full body HSPA4 ablation might have some limitations. Deletion of *Hspa4* during whole life span might affect embryogenesis and thus influence the myogenesis. Moreover, our mouse model experiences global *Hspa4* deletion in all cell types and possible unexplored functions of HSPA4 might therefore influence the outcome. Therefore, rescue study to address the ability of targeted HSPA4 expression using viral-mediated gene delivery with adeno-associated viral (AAV) vectors or non-viral nanoparticles delivery approach to correct the muscle phenotype in *Hspa4*-KO mice is warranted. Moreover, generation and characterization of muscle-specific *Hspa4*-KO mice are required to rule out the possibility of secondary effects.

In conclusion, we demonstrate that the deletion of HSPA4 in skeletal muscle leads to a progressive generalized myopathy, highlighting the critical role of HSPA4 in regulating the genetic repertoire required for the appropriate maintenance of skeletal muscle integrity. Furthermore, these findings support the investigation of HSPA4 as a novel therapeutic target for the amelioration of many inherited muscle diseases with impaired autophagy.

## Supplementary Information

**Additional file 1: Fig. S1** HSPA4 protein is ubiquitously expressed in different types of skeletal muscles. **Fig. S2** Maintained glucose and lipid metabolism in *Hspa4*-KO mice **Fig. S3**. Increased centrally nucleated myofibers in *Hspa4*-KO soleus muscle. **Fig. S4** Myopathy in the paraspinal muscle in 18-month-old *Hspa4*-KO mice. **Table S1**. List of mouse primers used in this study.

## Data Availability

All data generated or analyzed during this study are included in this article and its Additional Information.

## Abbreviations

AAV: Adeno-associated virus
ANOVA: Analysis of variance
CNF: Centrally nucleated fibers
CTX: Cardiotoxin
DM: Differentiation media
GC: Gastrocnemius muscle
GM: Growth media
DMEM: Dulbecco’s modified eagle’s medium
H&E: Hematoxylin and eosin
HSPA4: Heat shock protein A4
HSPs: Heat shock proteins
Il: Interleukins
KO: Knockout
LC3: Microtubule associated protein1 light chain 3
Myh3: Embryonic muscle myosin heavy chain
Myh8: Perinatal muscle myosin heavy chain
PBS: Phosphate-buffered saline
PQC: Protein quality control
qRT-PCR: Quantitative real-time polymerase chain reaction
TA: Tibialis anterior muscle
UPS: Ubiquitin–proteasome system
WT: Wild-type
