# Supplementary material for: Heat shock protein A4 ablation leads to skeletal muscle myopathy associated with dysregulated autophagy and induced apoptosis
Source: J Transl Med. 2022 May 14;20:229. doi: 10.1186/s12967-022-03418-3 (PMC9107738; doi:10.1186/s12967-022-03418-3)
Supplement: Supplementary file 1 — Additional file 1: Fig. S1 HSPA4 protein is ubiquitously expressed in different types of skeletal muscles. Fig. S2 Maintained glucose and lipid metabolism in Hspa4-KO mice Fig. S3. Increased centrally nucleated myofibers in Hspa4-KO soleus muscle. Fig. S4 Myopathy in the paraspinal muscle in 18-month-old Hspa4-KO mice. Table S1. List of mouse primers used in this study. [file 12967_2022_3418_MOESM1_ESM.pdf]

Additional file 1 for

Heat shock protein A4 ablation leads to skeletal muscle myopathy associated with dysregulated autophagy and induced apoptosis

Manar Elkenani, Amal Z. Barakat, Torsten Held, Daniel Marques Rodrigues, Sherok Mobarak, Surabhi Swarnkar, Ibrahim M. Adham, Belal A. Mohamed

The file includes:

Supplementary figure legends

Fig. S1. HSPA4 protein is ubiquitously expressed in different types of skeletal muscles.

Fig. S2. Maintained glucose and lipid metabolism in *Hspa4*-KO mice

Fig. S3. Increased centrally nucleated myofibers in *Hspa4*-KO soleus muscle.

Fig. S4. Myopathy in the paraspinal muscle in 18-month-old *Hspa4*-KO mice.

Supplementary figures

Supplementary table S1 List of mouse primers used in this study

Fig. S1. HSPA4 protein is ubiquitiously expressed in different types of skeletal muscles. Representative immunoblot of HSPA4 protein in skeletal muscles. Dia, Diaphragm; GC, Gastrocnemius; TA, Tibialis anterior.

Fig. S2. Maintained glucose and lipid metabolism in *Hspa4*-KO mice. (A) Serum glucose levels in 2-week-old WT and *Hspa4*-KO mice. (B and C) Northern blot (B) and real time PCR (C) analyses of gluconeogenic enzyme expression, phosphoenolpyruvate carboxykinase 1 (*Pepck)*, in 2-month-old liver. (D and E) Northern blotting (D) and real time PCR (E) of hepatic lipid transport related-genes expression, *Sar1b* and *ApoB* in 10-day-old mice. (E) Real time PCR analyses of hepatic *Sar1b* and *ApoB* in 10-day-old mice. (F) Northern blotting analyses of growth hormone-responsive gene expression, solute carrier organic anion transporter (*Slco1b2),* in 10- and 14-day-old liver. (G) *Slco1b2* expression in 14-day-old liver, assessed by real time PCR. Data are expressed as mean ± SEM, two-tailed unpaired Student's t-test. Numbers within columns indicate mice.

Fig. S3. Increased centrally nucleated myofibers in *Hspa4*-KO soleus muscle. (A) H&E stained-sections of soleus from 3-week-old mice. (B) Increased centrally nucleated fibers in soleus muscle at 2- and 3-week-old *Hspa4*-KO mice.

Fig. S4. Myopathy in the paraspinal muscle in 18-month-old *Hspa4*-KO mice. Note centrally nucleated myofibers (arrows).

Fig. S1

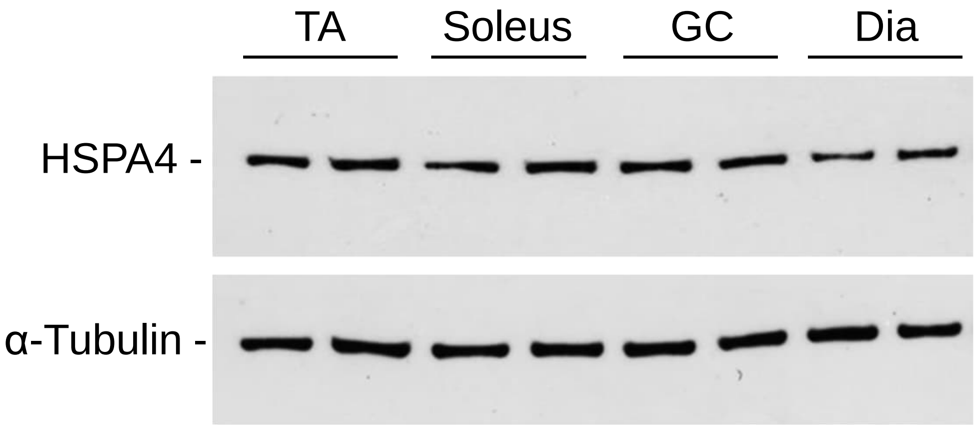

Fig. S2

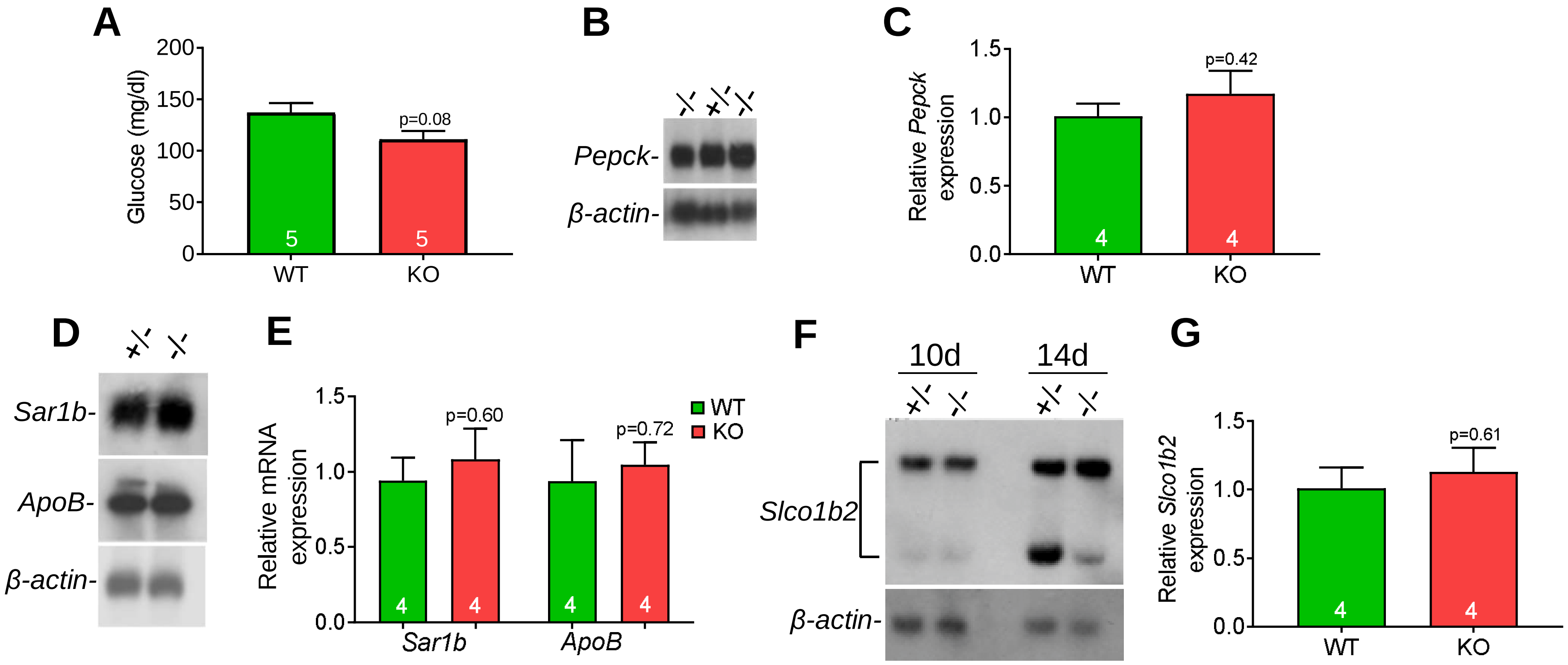

A
Glucose (mg/dl)
200
150
100
50
0
p=0.08
5
5
WT
KO
B
-/-
+/-
-/-
Pepck-
β-actin-
C
Relative Pepck expression
1.5
1.0
0.5
0.0
p=0.42
4
4
WT
KO
D
+/-
-/-
Sar1b-
ApoB-
β-actin-
E
Relative mRNA expression
1.5
1.0
0.5
0.0
p=0.60
p=0.72
WT
KO
4
4
4
4
Sar1b
ApoB
F
10d
14d
+/-
-/-
+/-
-/-
Slco1b2
β-actin-
G
Relative Slco1b2 expression
1.5
1.0
0.5
0.0
p=0.61
4
4
WT
KO

Fig. S3

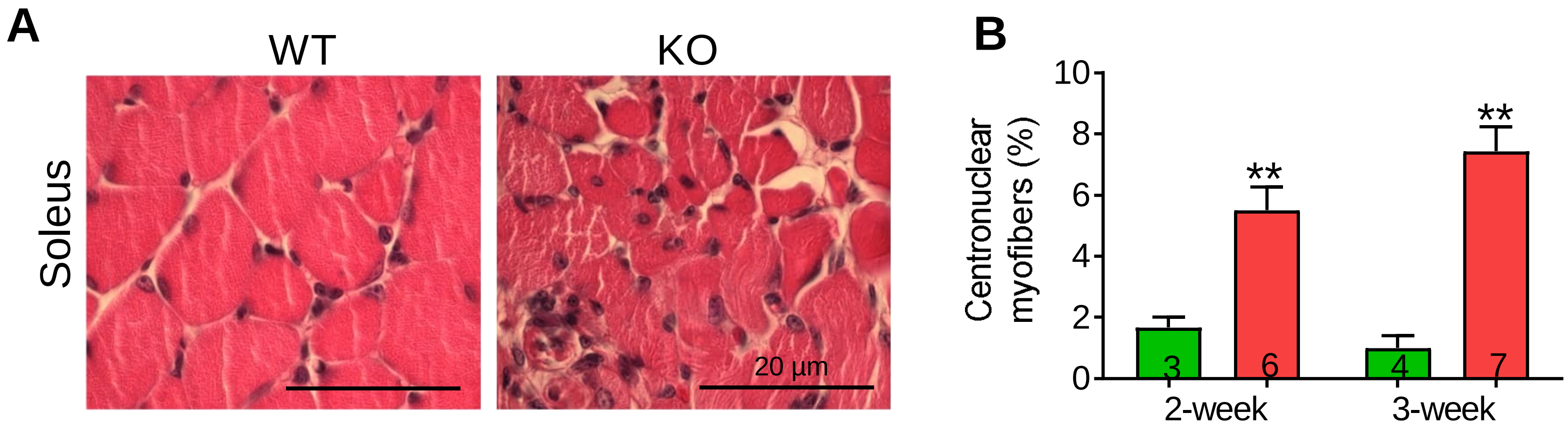
A
WT
KO
Soleus
20 µm
B
Centronuclear myofibers (%)
10
8
6
4
2
0
**
**
3
6
4
7
2-week
3-week

Fig. S4

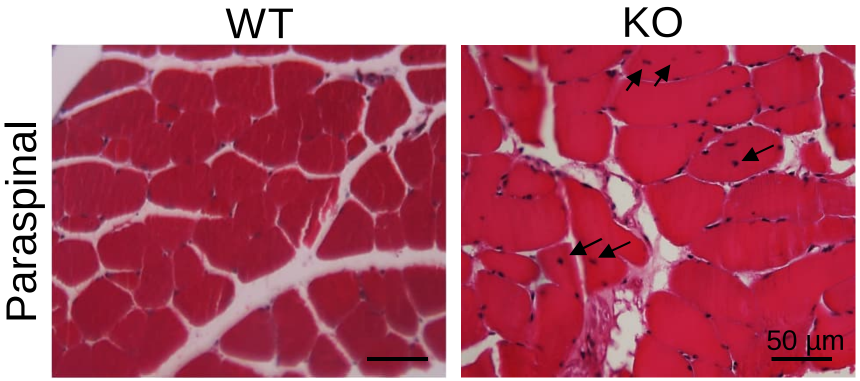

Table S1. List of mouse primers used in this study

**Real time PCR**	
Hprt1	F: 5`-AGCCCCAAAATGGTTAAGGTTGC-3`<br>R: 5`-TTGCAGATTCAACTTGCGCTCAT-3`
Myh8	F: 5`-AACAGAAACGCAATGCTGAGG-3`<br>R: 5`-TCGCCTGTAATTTGTCCACCA-3`
Myh3	F: 5`-AAAAGGCCATCACTGACG-3`<br>R: 5`-CAGCTCTCTGATCCGTGTCTC-3`
MAFbx	F: 5'-CTCTGTACCATGCCGTTCCT-3'<br>R: 5'-GGCTGCTGAACAGATTCTCC-3'
MuRF1	F: 5'-ACGAGAAGAAGAGCGAGCTG-3'<br>R: 5'-CTTGGCACTTGAGAGGAAGG-3'
Pepck	F: 5'-AGCCTGCTCCAGCTTTGA-3'<br>R: 5'-CCCTAGCCTGTTCTCTGTGC-3'
Sar1b	F: 5'-CAGCACGTCCCAACACTACA-3'<br>R: 5'-AAACGTCATGCCAGCAATAGT-3'
ApoB	F: 5'-TGGGATTCCATCTGCCATCTCGAG-3'<br>R: 5'-GTACAGATCCATCACAGGACAATG-3'
Myod1	F: 5'-GCGCTCCAACTGCTCTGATG-3'<br>F: 5'-TCGACACAGCCGCACTCTTC-3'
Myogenin	F: 5'-CCAACCCAGGAGATCATTTG-3'<br>R: 5'-GGAAGGCAACAGACATATCCTC-3'
Pax7	F: 5'-GGGATGTTCAGCTGGGAAA-3'<br>R: 5'-CACTCGGCTAATCGAACTCA-3'
Cd68	F: 5'-*ACCGCTTATAGCCCAAGGAAC*-3'<br>R: 5'-*CGTGAAGGATGGCAGGAGAGTAA*-3'
Il1b	F: *5'-ATGCCACCTTTTGACAGTGATG-3'*<br>R: *5'-GCTCTTGTTGATGTGCTGCT-3'*
Il6	F: *5'-TCGTGGAAATGAGAAAAGAGTTGTG-3'*<br>R: *5'-ATCCAGTTTGGTAGCATCCATCA-3'*
F4/80	*F: 5'-CCCCAGTGTCCTTACAGAGTG-3'*<br>*R: 5'-GTGCCCAGAGTGGATGTCT-3'*
**Northern blotting**	
Pepck	F 5`- GTGGAGGAGATCGACAGGTATC -3'<br>5`- CTCACACAGAGACACGTTCAC-3'
ApoB	F 5`- CCAACAGAAATGTCCACAGAGA -3'<br>R 5`- AGCCACTGGAGGATGTGAGTAT -3'

Slco1b2	F 5`-TGCGATGGATTCAGGATATT-3'<br>R 5`-ACCCCTTTTCACAACCTTTC -3'
